# Structure-dependent recruitment and diffusion of guest proteins in liquid droplets of FUS

**DOI:** 10.1038/s41598-022-11177-w

**Published:** 2022-05-02

**Authors:** Kiyoto Kamagata, Nanako Iwaki, Saori Kanbayashi, Trishit Banerjee, Rika Chiba, Virginie Gaudon, Bertrand Castaing, Seiji Sakomoto

**Affiliations:** 1 Institute of Multidisciplinary Research for Advanced Materials, Tohoku University, Katahira 2-1-1, Aoba-ku, Sendai, 980-8577 Japan; 2 Department of Chemistry, Graduate School of Science, Tohoku University, Sendai, 980-8578 Japan; 3 Graduate School of Life Sciences, Tohoku University, Katahira 2-1-1, Aoba-ku, Sendai, 980-8577 Japan; 4 Centre de Biophysique Moléculaire, CNRS, UPR4301, rue Charles Sadron, 45072 Orléans, France; 5 Department of Synthetic Chemistry and Biological Chemistry, Graduate School of Engineering, Kyoto University, Katsura, Nishikyo-ku, Kyoto, 615-8510 Japan

**Keywords:** Intrinsically disordered proteins, Single-molecule biophysics

## Abstract

Liquid droplets of a host protein, formed by liquid–liquid phase separation, recruit guest proteins and provide functional fields. Recruitment into p53 droplets is similar between disordered and folded guest proteins, whereas the diffusion of guest proteins inside droplets depends on their structural types. In this study, to elucidate how the recruitment and diffusion properties of guest proteins are affected by a host protein, we characterized the properties of guest proteins in fused in sarcoma (FUS) droplets using single-molecule fluorescence microscopy in comparison with p53 droplets. Unlike p53 droplets, disordered guest proteins were recruited into FUS droplets more efficiently than folded guest proteins, suggesting physical exclusion of the folded proteins from the small voids of the droplet. The recruitment did not appear to depend on the physical parameters (electrostatic or cation–π) of guests, implying that molecular size exclusion limits intermolecular interaction-assisted uptake. The diffusion of disordered guest proteins was comparable to that of the host FUS, whereas that of folded proteins varied widely, similar to the results for host p53. The scaling exponent of diffusion highlights the molecular sieving of large folded proteins in droplets. Finally, we proposed a molecular recruitment and diffusion model for guest proteins in FUS droplets.

## Introduction

Membraneless organelles, such as stress granules and nucleoli, are liquid droplets formed by liquid–liquid phase separation (LLPS)^1–5^. The droplets recruit various LLPS-relevant molecules, including proteins and RNA, and maintain their movements inside them, triggering a series of continuous biological reactions. LLPS-relevant proteins such as LAF-1, fused in sarcoma (FUS), TDP-43, and p53 have recently been identified. They are categorized as host (or scaffold) and guest (or client) proteins. Host proteins can form liquid droplets, whereas guest proteins are recruited to the liquid droplets. For host proteins, intrinsically disordered proteins (IDPs), rather than folded proteins, tend to form droplets via multivalent intermolecular interactions, such as cation–π, hydrophobic, and electrostatic interactions^6–12^. Despite the serial discovery of LLPS-relevant proteins and the biophysical characterization of host proteins, the molecular grammar of LLPS of guest proteins remains poorly understood.

In our previous study, we investigated the recruitment properties of 18 guest proteins in liquid droplets of host p53 using fluorescence microscopy^13^. A series of data showed similar recruitment properties between folded and disordered guest proteins, which were primarily dictated by cation–π and electrostatic interactions. The importance of cation–π interactions in recruitment was also found in the droplets of Ddx4^14^ and FUS^9^. However, this similar recruitment property between folded and disordered guest proteins to p53 droplets differ from the preferential tendency of IDPs to induce LLPS as hosts rather than folded proteins^6,15^. In addition, guest proteins differ among membraneless organelles^3^. Considering these facts, the recruitment property depends on the microscopic droplet characteristics of host proteins, such as intermolecular interactions and void exclusion from droplets. In fact, FUS, used as a host protein in this study, forms droplets mainly through cross-β structures^16–18^ and cation–π interactions^9,19^, whereas p53 forms droplets via electrostatic interactions. In addition, the voids formed by the non-uniform distribution of host molecules^20–22^ might exclude large guest proteins from the droplets, which has not been observed in p53 droplets.

Unlike similar recruitment property between folded and disordered guest proteins, the translational dynamic properties of guest proteins, determined by single-molecule fluorescence microscopy, depend on the guest protein structure in p53 droplets^13^. IDPs exhibit homogeneous slow diffusion, whereas folded proteins exhibit a wide range of diffusion depending on their size and intermolecular interactions. The diffusion properties of guest proteins might be affected by the microscopic complex and dynamic network of host proteins in the droplet. In particular, the diffusion of guest proteins is slowed down by tight intermolecular interactions and physical restriction inside the small voids of droplets.

In this study, we aimed to clarify how host proteins affect the recruitment and diffusion properties of guest proteins. We chose FUS as the host protein because it is a well-studied LLPS-relevant protein^9,10,16–19,23–25^ and has different physical characteristics from p53, such as an oligomeric state (monomer for FUS and tetramer for p53), LLPS-relevant interaction (see above), and molecular size (526 residues for FUS and 1,572 residues for p53 tetramer). We characterized the recruitment and diffusion properties of guest proteins of different sizes, structures, and oligomeric states in liquid droplets of FUS using single-molecule fluorescence microscopy. These properties of FUS droplets were compared with those of p53 droplets^13^. We found similar diffusion properties but different recruitment properties for folded and disordered guest proteins in the two droplets.

## Results

### Disordered proteins are highly recruited to FUS droplets

**Figure 1:**
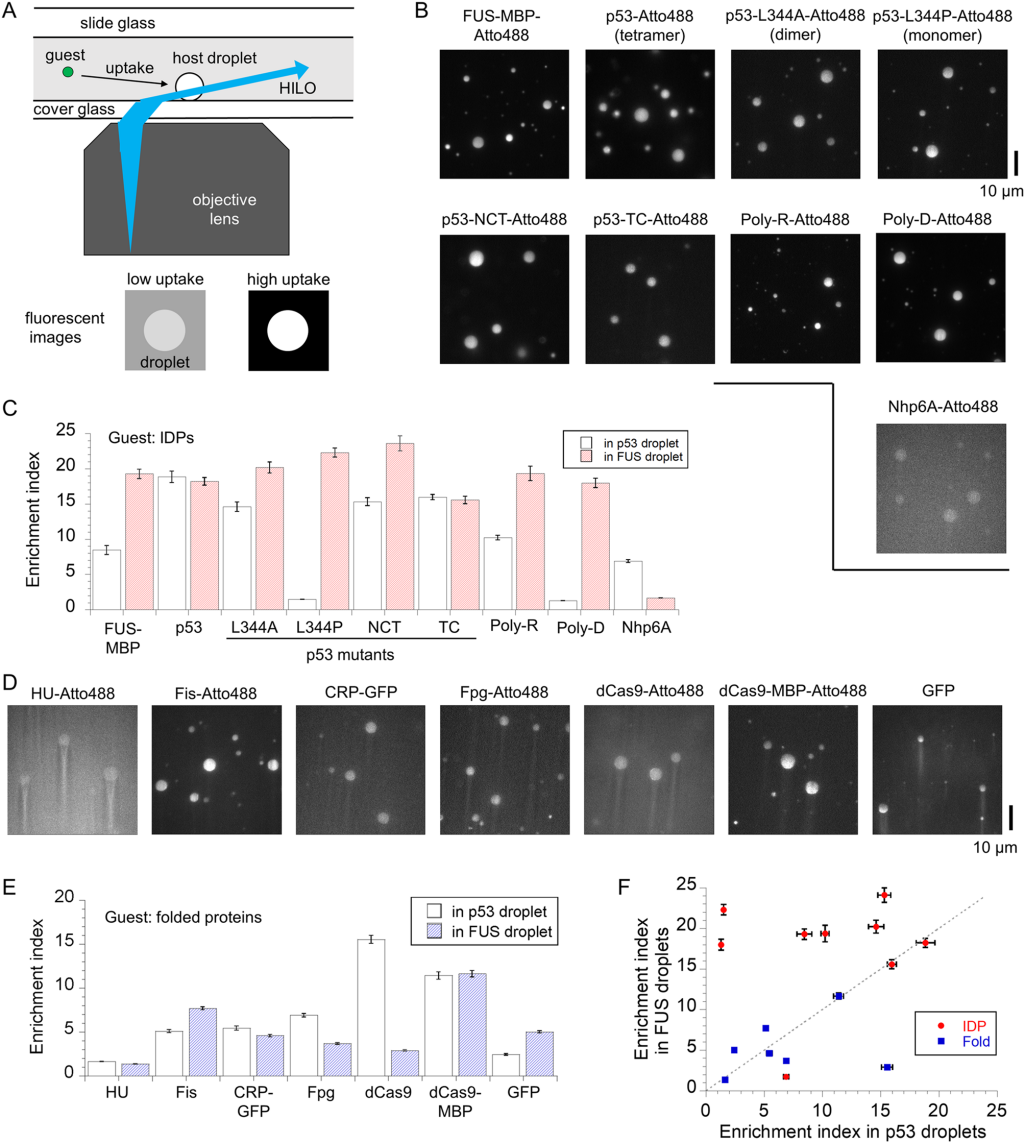
Recruitment property of guest proteins in liquid FUS droplets and comparison with that in liquid p53 droplets. (**A**) Schematic diagram of fluorescent microscopic measurements with highly inclined and laminated optical sheet (HILO) illumination for labeled guest proteins in non-labeled host FUS droplets. Bottom represents schematic fluorescent images of guest uptake. (**B**) Fluorescent images of Atto488-labeled intrinsically disordered proteins (IDPs) and artificial polymers in non-labeled FUS droplet solution. (**C**) Enrichment index (EI) of the guest IDPs and artificial polymers into FUS or p53 droplets. (**D**) Fluorescent images of labeled folded proteins, CRP-GFP, and GFP in non-labeled FUS droplet solution. (**E**) EI of the guest folded proteins into FUS or p53 droplets. (**F**) Comparison of EI in between p53 and FUS droplets. The grey dashed line (EI_FUS_ = EI_p53_) is displayed as a guide for eye. The EI values in p53 droplets were plotted from the Ref.^13^. In panels (**C**), (**E**), and (**F**), bars and errors represent the mean and standard errors for the average EIs of each droplet, respectively.

We examined the recruitment of guest proteins, labeled with a fluorescent dye or fused to GFP, into non-labeled FUS droplets using fluorescence microscopy with highly inclined and laminated optical sheet (HILO) illumination^13^ (Fig. 1A). For the host, we used FUS conjugated to a soluble maltose-binding protein (MBP) tag (FUS-MBP) without cleavage of the MBP tag, as FUS-MBP forms stable liquid droplets in the presence of a molecular crowder dextran, which minimizes the conversion of the liquid droplets to hydrogels in MBP tag-cleaved FUS^26^. We used the same sets of guest proteins as those reported for p53 droplets^13^ for comparison between the two hosts. Briefly, the guest proteins having a wide range of sizes, folded or disordered structures, and different oligomeric states were tested to extract the recruitment and diffusion rules in FUS droplets. No specific interactions between FUS and guests were assumed under no droplet condition, since the guest proteins, except p53, originate from other species and no significant interaction between FUS and p53 has been shown^13^. p53 has been reported to be recruited into FUS droplets in vivo^27^. For fluorescence detection, we used only Atto488 as the fluorescent dye to minimize the effect of a dye on uptake (Supplementary Fig. S1).

We first examined Atto488-labeled FUS-MBP uptake into non-labeled FUS droplets (Fig. 1B). The concentrations were set to 0.1 μM for labeled guests and 10 μM for the non-labeled host. The average enrichment index (EI), calculated by dividing the fluorescence intensity in the droplets by that in the solution, was 19.3, reflecting high uptake of the host protein (Fig. 1C, Table 1). Next, we examined the uptake of the Atto488-labeled p53 tetramer, a droplet-forming protein, into FUS droplets. The p53 tetramer showed similarly high recruitment (average EI = 18.2), consistent with localization of p53 in FUS droplet in vivo^27^. To examine the effect of oligomeric states of p53 on FUS droplet uptake, we measured Atto488-labeled p53 dimer (L344A)^28^ and monomer (L344P)^29–31^ mutants (Fig. 1B). The average EI of the p53 dimer and monomer mutants increased slightly to 20.2 and 22.3, respectively. The slightly reduced uptake of p53 into FUS droplets with increased oligomeric states was different from the increased uptake of p53 into p53 droplets^13^ (Fig. 1C). In particular, the high recruitment of the p53 monomer in FUS droplets was largely different from its low one in p53 droplets^13^. We also investigated the effect of removing either the droplet-forming disordered domains (N- and C-terminal domains) of p53 on its uptake into FUS droplets. NCT and TC mutants of p53, which lack the C-terminal domain and N-terminal plus core domains, respectively, while maintaining a tetramer^32–34^, showed high recruitment into FUS droplets (Fig. 1B,C; Table 1). Thus, droplet-forming proteins showed high recruitment to FUS droplets.

**Table 1:** Enrichment indices, physical parameters, and diffusion coefficients of guest proteins in FUS and p53 droplets.

Guest proteins	FUS droplets		p53 droplets*		Length (AA)
	Enrichment index	*D* (μm^2^ s^−1^)	Enrichment index	*D* (μm^2^ s^−1^)	
**IDPs**					
FUS-MBP	19.3 ± 0.7	0.074 ± 0.007	8.5 ± 0.6	0.051 ± 0.005	934
p53 tetramer	18.2 ± 0.6	0.021 ± 0.002	18.9 ± 0.8	0.031 ± 0.002	1572
p53 L344A (dimer)	20.2 ± 0.8	0.049 ± 0.005	14.6 ± 0.7	0.039 ± 0.002	786
p53 L344P (monomer)	22.3 ± 0.6	0.065 ± 0.006	1.51 ± 0.04	0.046 ± 0.004	393
p53 NCT (tetramer)	24.1 ± 0.9	0.034 ± 0.004	15.3 ± 0.6	0.026 ± 0.003	1452
p53 TC (tetramer)	15.6 ± 0.5	0.065 ± 0.004	16.0 ± 0.4	0.054 ± 0.006	408
Nhp6A	1.71 ± 0.03	0.046 ± 0.009	6.9 ± 0.2	0.048 ± 0.004	93
poly-Arg	19 ± 1	0.046 ± 0.006	10.2 ± 0.3	0.036 ± 0.003	200
poly-Asp	18.0 ± 0.7	0.060 ± 0.006	1.31 ± 0.02	0.052 ± 0.003	200
**Folded proteins**					
HU (dimer)	1.37 ± 0.02	1.51 ± 0.07	1.63 ± 0.02	0.43 ± 0.06	184
Fis (dimer)	7.7 ± 0.2	0.44 ± 0.03	5.1 ± 0.2	0.93 ± 0.08	196
Fpg	3.7 ± 0.1	0.93 ± 0.08	6.9 ± 0.2	1.31 ± 0.09	272
GFP	5.0 ± 0.1	0.54 ± 0.04	2.4 ± 0.1	1.67 ± 0.08	246
CRP-GFP (dimer)	4.6 ± 0.1	0.045 ± 0.004	5.5 ± 0.2	0.19 ± 0.02	912
dCas9	2.9 ± 0.1	0.040 ± 0.003	15.6 ± 0.5	0.070 ± 0.004	1371
dCas9-MBP	11.6 ± 0.4	0.032 ± 0.004	11.4 ± 0.4	0.048 ± 0.003	1779

*Data in the p53 droplets were obtained from Kamagata et al.^13^.

To confirm whether other IDPs, but not forming droplets by themselves, are similarly recruited into the droplets, we examined the uptake of charge-rich IDPs and polymers into FUS droplets (Fig. 1B,C). The positively charged Atto488-labeled poly-R peptide (with a median of 200 residues) showed high recruitment (average EI = 19). In addition, the negatively charged Atto488-labeled poly-D peptide (with a median of 200 residues) exhibited high recruitment (average EI = 18.0). The relative recruitment capability of poly-R and poly-D in IDP series was larger than that in p53 droplets^13^ (Fig. 1C). In contrast, the average EI of the Atto488-labeled DNA-binding protein Nhp6A (N-terminal disordered region and globular HMGB domain) decreased to 1.71. The low recruitment of Nhp6A in FUS droplets differed from the moderate one in p53 droplets^13^ (Fig. 1C). Taken together, IDPs, except for small Nhp6A, were highly recruited into FUS droplets.

### Folded proteins show low to moderate uptake properties in FUS droplets

As the uptake property of IDPs is modulated by the host protein, that of folded proteins may depend on the host protein. Next, we investigated the uptake of several charge-rich folded proteins into FUS droplets (Fig. 1D,E, Table 1). Atto488-labeled dimeric DNA-binding proteins, HU and Fis, showed low and moderate recruitment, respectively (average EI = 1.71 and 7.7 for HU and Fis, respectively). Dimeric DNA-binding cAMP receptor protein (CRP) conjugated to GFP exhibited low recruitment (average EI = 4.6). In addition, the Atto488-labeled monomeric DNA-binding protein, Fpg, was slightly recruited (average EI = 3.7). The Atto488-labeled monomeric DNA-binding protein, dCas9 (a deactivated mutant lacking the ability to cleave DNA), showed low recruitment (average EI = 2.9), which differed from its high recruitment in p53 droplets^13^ (Fig. 1E). In contrast, the average EI of Atto488-labeled MBP-conjugated dCas9 (dCas9-MBP) was 11.6, comparable to its high recruitment in the p53 droplets^13^ (Fig. 1E, Table 1). Furthermore, the fluorescent protein GFP exhibited low uptake (average EI = 5.0). The recruitment pattern of series of folded proteins in FUS droplets, except for dCas9, varied slightly from that in p53 droplets^13^ (Fig. 1E). Taken together, the EI values of folded proteins in the FUS droplets were distributed in low to moderate ranges.

To compare the uptake properties of guest proteins by p53 and FUS droplets, we plotted the average EIs of the two hosts (Fig. 1F). Large deviations were observed from the proportional relationship, particularly for IDPs, resulting in partly similar but different uptake properties between the two hosts.

### Size-independent uptake is caused by a trade-off between intermolecular interactions and size exclusion from droplet voids

**Figure 2:**
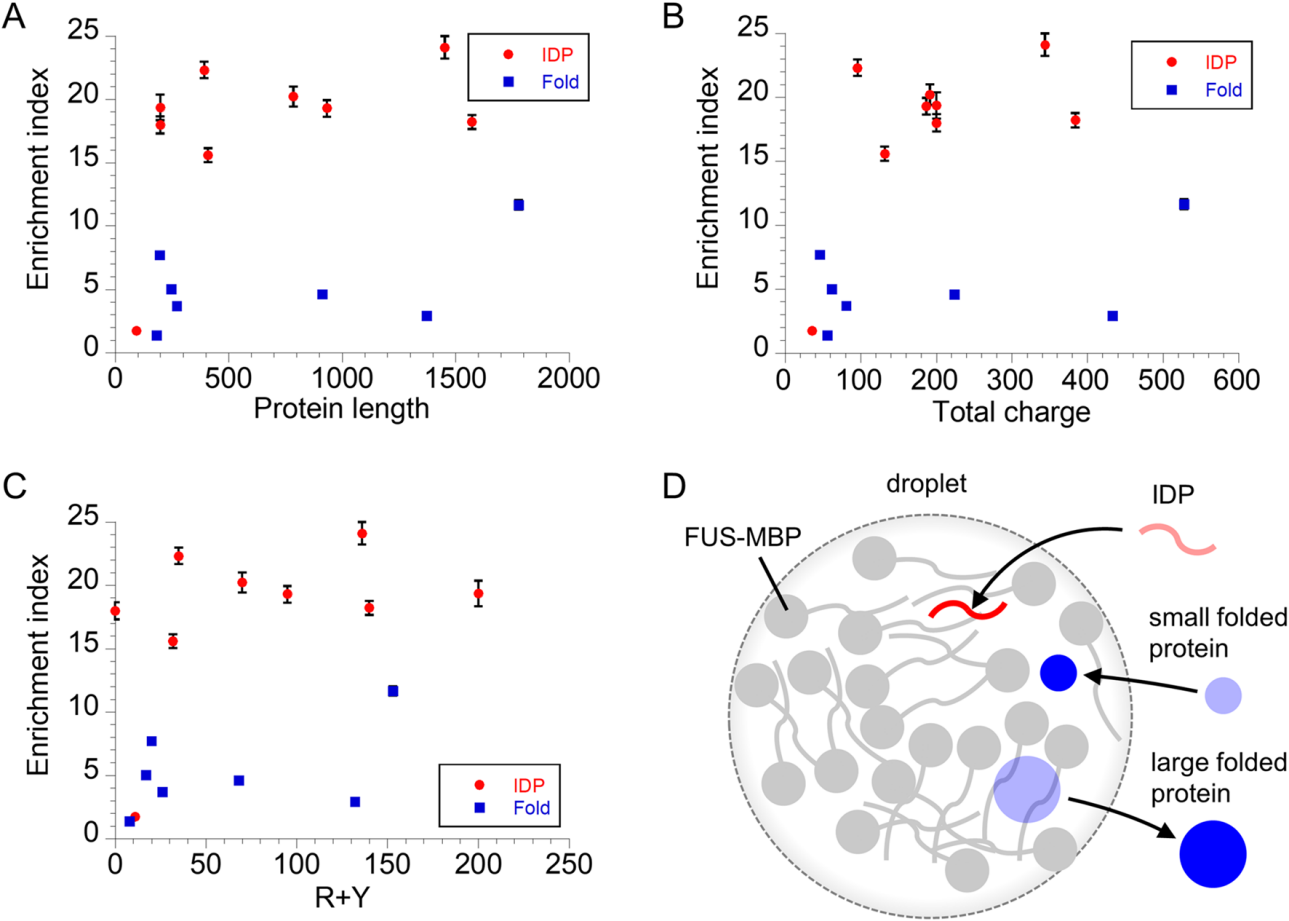
Enrichment index of guests in FUS droplets depends on the structural type, rather than intermolecular interaction. (**A**) Protein length dependence of enrichment index (EI) of guest proteins. (**B**) Total charge dependence of EI of guest proteins. (**C**) EI of guest proteins against a function of R plus Y number. In panels (**A**–**C**), errors denote the standard errors. (**D**) Proposed model of structure-dependent recruitment in a FUS droplet. The droplet (dashed grey circle) is formed by host FUS-MBP (grey). IDP (red) and small folded protein (small blue circle) are recruited into the droplet void, whereas large folded protein (large blue circle) is excluded from the relatively small void of the droplet.

The uptake capability of p53 droplets was moderately correlated with guest protein size, electrostatic interactions between the host and guests, and cation–π interactions between the host and guests^13^. To clarify whether this relationship is true for FUS droplets, we compared the EI values for FUS droplets and three physical parameters of guest proteins: protein length, total charge number (R, K, D, and E), and number of R plus Y residues^9^ (Fig. 2A–C). Unlike p53 droplets, the EI values in FUS droplets, except for Nhp6A, were not dependent on these three parameters. Furthermore, the EI values in FUS droplets did not correlate with the net charge of guest proteins and the total charge of solvent-exposed residues of guest proteins, suggesting that the uptake capability is not simply explained by the electrostatic interactions of net charges between host and guest molecules (Supplementary Fig. S2A,B). This was also supported by no correlation of the EI values of monomeric IDPs or artificial polymers in FUS droplets with joint sequence charge decoration reflecting sequence-specific polyelectrostatic interactions between host and guest disordered regions^35^ (Supplementary Fig. S2C). These facts imply that even if intermolecular interactions increase in large proteins, the recruitment ability is masked by other factors (e.g., exclusion from voids in droplets). In p53 droplets, no significant differences in uptake were observed between folded and disordered proteins^13^. In contrast, IDPs were preferentially recruited into FUS droplets rather than folded proteins (Fig. 2A–C). Considering this high recruitment tendency of IDPs compared to folded proteins and the 1.7-fold smaller molecular size of host FUS compared to the host p53 tetramer, the average void size in FUS droplets is likely smaller than that in the p53 tetramer droplet. IDPs can adapt their structures into voids, but large folded proteins are excluded from the relatively small voids owing to their unchanged structure (Fig. 2D). Accordingly, void exclusion would compensate for the large intermolecular interactions expected in large folded proteins, resulting in the size-independent uptake of folded proteins. The voids in FUS droplets were supported by the pressure-mediated dissolution of droplets^36^. Overall, the apparent size-independent uptake in the FUS droplets is interpreted as a trade-off between intermolecular interactions and size exclusion from droplet voids (see details in “Discussion” section).

### Diffusion dynamics of guest proteins in FUS droplets depend on the structural properties of guest molecules

**Figure 3:**
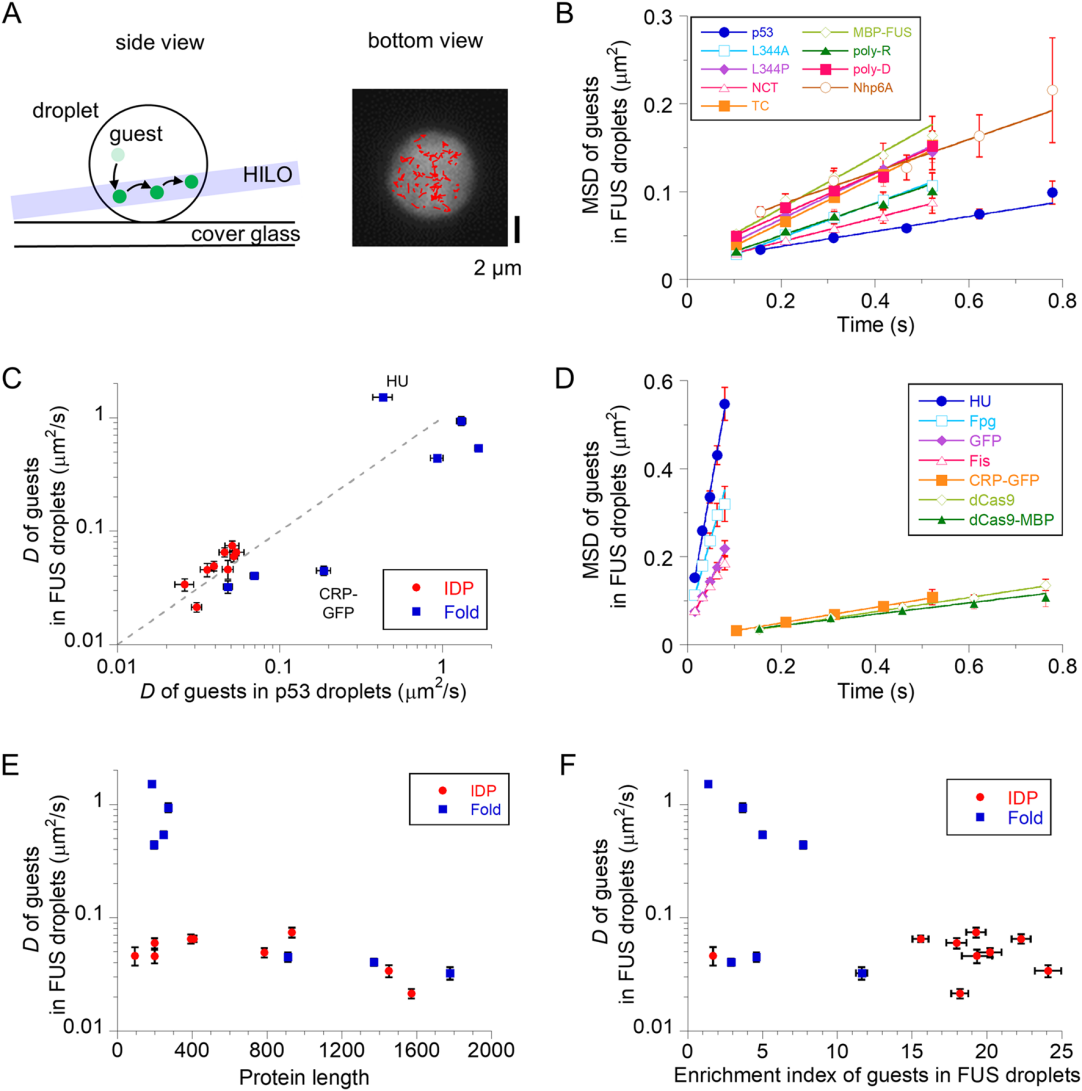
Translational diffusion of guests in FUS droplets depend on the structural type. (**A**) Schematic diagram of single-molecule tracking of fluorescent guests in a droplet (side view) and typical trajectories of guest FUS-MBP molecules in a non-labeled FUS droplet (bottom view). In the left panel, highly inclined and laminated optical sheet (HILO) illumination minimizes background fluorescence, enabling single-molecule detection of guests (green) in droplets. In the right panel, the typical trajectories of single molecules (red) are overlaid in time-averaged fluorescent images (white droplet). (**B**) Mean square displacement (MSD) plots of the labeled intrinsically disordered proteins (IDPs) and artificial polymers in non-labeled FUS droplets. (**C**) Two dimensional plot of diffusion coefficient (*D*) of the guest proteins into p53 versus FUS droplets. The data in p53 droplets were plotted from the Ref.^13^. The grey dashed line (*D*_FUS_ = *D*_p53_) is displayed as a guide for eye. (**D**) MSD plots of labeled folded proteins, CRP-GFP, and GFP in non-labeled FUS droplets. (**E**) Protein length dependence of the average *D* of guest proteins in FUS droplets. (**F**) Enrichment index (EI) dependence of the average *D* of guest proteins in FUS droplets. In all panels except for (**A**), errors of EI and *D* denote the standard and fitting errors, respectively.

To clarify the translational dynamics of labeled guest proteins in non-labeled FUS droplets, we performed single-molecule tracking measurements as reported previously^13^. For single-molecule detection, the concentrations of labeled guests were reduced to 0.1–0.5 nM. We first measured the dynamics of FUS-MBP-Atto488. We tracked the centers of the bright spots inside the droplets (Fig. 3A). The dynamic properties were analyzed using mean square displacement (MSD) plots obtained from the trajectories. The MSD plots revealed a linear relationship with time interval, indicating that FUS molecules diffused inside the droplets (Fig. 3B). The average diffusion coefficient (*D*), obtained by fitting the MSD plots to a linear equation with a 4*D* slope, was 0.074 μm^2^/s.

To elucidate the dynamic properties of other guest proteins in FUS droplets, we measured several Atto488-labeled IDPs, including p53 mutants and artificial polymers. In all cases, the linear MSD plots confirmed diffusion within the droplets (Fig. 3B). The average *D* values of the p53 oligomer mutants ranged from 0.021 (tetramer) to 0.065 (monomer) μm^2^/s (Table 1). In addition, NCT and CT mutants of p53, lacking either droplet-forming disordered domains, showed relatively slow diffusion (Table 1). Accordingly, the diffusion of guest p53 is less sensitive to the reduction of the oligomeric state or droplet-forming disordered domains. The average *D* value of Nhp6A, irrespective of low uptake, was 0.046 μm^2^/s, which is slightly lower than that of host FUS. In addition, poly-R and poly-D diffused equally slowly (Table 1). The slow diffusion property of IDPs in FUS droplets was similar to that in p53 droplets (Fig. 3C).

Next, we investigated the dynamics of several Atto488-labeled or GFP-fused folded proteins in FUS droplets. The linear MSD plots for all folded samples confirmed diffusion within the droplets (Fig. 3D). In contrast to IDPs, HU-Atto488, Fis-Atto488, Fpg-Atto488, and GFP showed linear MSD plots with large slopes. The average *D* values were more than 5.9-fold larger than those of FUS and widely distributed from 0.44 to 1.51 μm^2^/s (Table 1). In addition, the *D* value of each molecule was distributed widely in the range between 0 and 4.5 μm^2^/s, suggesting heterogeneous intermolecular interactions in the droplets (Supplementary Fig. S3). In contrast, CRP-GFP, dCas9-Atto488, and dCas9-MBP-Atto488 diffused slowly on average, comparable to IDPs (Table 1). The overall diffusion property of folded proteins in FUS droplets was similar to that in p53 droplets, except for HU and CRP-GFP, which showed relatively large deviations (Fig. 3C).

The average *D* plots against protein length indicated that the diffusion of guest IDPs was almost comparable to that of host FUS, irrespective of the wide range of protein lengths (Fig. 3E). In contrast, folded proteins diffused in a size-dependent manner. The diffusion of folded proteins was faster than that of IDPs within 300 residues, and became comparable to that of IDPs above 800 residues (Fig. 3E). CRP-GFP diffused relatively slowly in FUS droplets compared to that in p53 droplets, suggesting that the small size of voids in FUS droplets restricted the moving space of CRP-GFP, which slowed the diffusion.

### Relationship between diffusion dynamics and recruitment in FUS droplets

To characterize this relationship, we plotted the data as a function of the EI and *D* values of FUS droplets (Fig. 3F). Folded proteins showed EI values concentrated in the low range, but the *D* values were widely distributed. In contrast, the EIs and *D* values of IDPs were mostly distributed in the high and low range, respectively. The two-dimensional plot clearly suggested that the structure of guest proteins determined their uptake and dynamic properties in FUS droplets. Compared to the plots of p53 droplets (Fig. 5D in Ref.^13^), the data of folded proteins in FUS droplets shifted to lower EI values, whereas those of IDPs moved to larger EI values. This structure-selective uptake of FUS droplets is likely caused by physical exclusion from the relatively small voids of FUS droplets.

## Discussion

Fluorescence measurements of the guest protein series in FUS droplets indicated that disordered guest proteins have a larger recruitment capability than folded guest proteins, which differs from the similar recruitment property between folded and disordered guest proteins in p53 droplets^13^. This different recruitment property between folded and disordered guest proteins in FUS droplets was attributed to the voids formed in the network of host FUS molecules inside the droplets. The voids in FUS droplets have been confirmed^36^. When the molecular size of the folded guest protein is larger than the size of the void, the guest protein should be excluded from the void (Fig. 2D). In contrast, even if the molecular weight of IDPs is the same as that of folded proteins, flexible IDPs can adapt their structures into the void and remain in it (Fig. 2D). Accordingly, IDPs are recruited into droplets more efficiently than folded proteins. Similar size-dependent exclusion of folded proteins has been observed in other systems^20,21^. Furthermore, this is consistent with the tendency of IDPs to form droplets as host proteins^6,15^. The different recruitment patterns between FUS and p53 droplets indicated that the average void size in p53 droplets was significantly larger than that in FUS droplets, reducing the size exclusion effect in p53 droplets.

The other key factor for recruitment is the intermolecular interaction of a guest protein with neighboring host molecules. Wang et al. reported that the number of R and Y residues of various FUS family proteins, corresponding to cation–π interactions, was correlated with the recruitment tendency in FUS droplets^9^. Contrary to this relationship, we did not observe any correlation between EI and cation–π or electrostatic interactions (Fig. 2C). Considering the effect of intermolecular interactions on molecular recruitment in droplets^9,13,37,38^, we conclude that intermolecular interactions are cancelled out by the physical exclusion of voids in FUS droplets.

**Figure 4:**
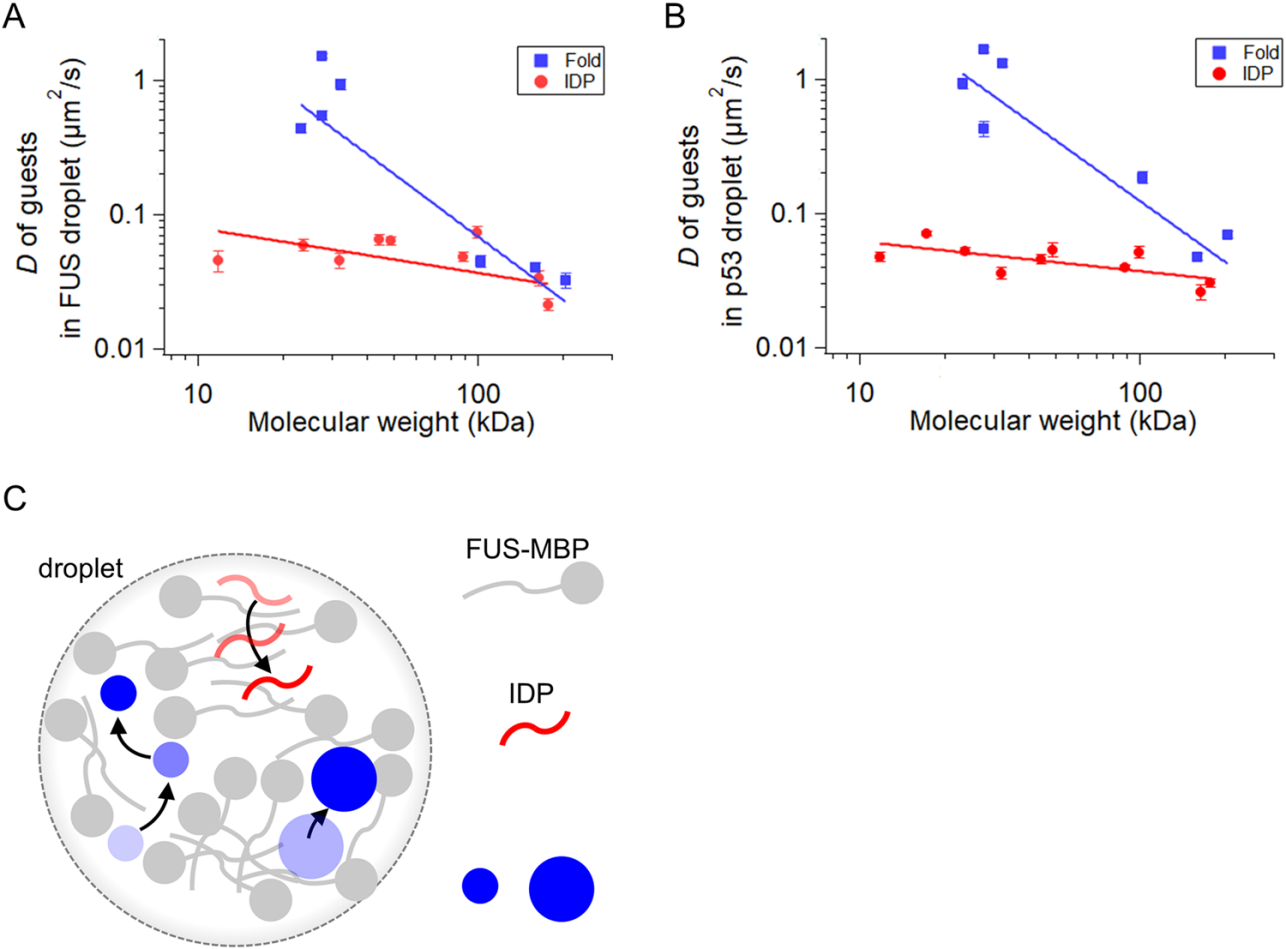
Scaling of diffusion of guests in droplets and diffusion model. (**A**,**B**) Diffusion of guest proteins in FUS (**A**) and p53 (**B**) droplets as a function of molecular weight. Blue and red plots represent the data for folded proteins and intrinsically disordered proteins (IDPs), respectively. Solid lines are best-fitted ones of a power-law dependence. The molecular weight of guest proteins, obtained using Expasy, includes that of fluorescent dyes. The *D* values in p53 droplets were plotted from the Ref.^13^. (**C**) Proposed model of structure-dependent diffusion in a FUS droplet. The droplet (dashed grey circle) is formed by host FUS-MBP (grey). IDP (red) diffuses slowly while interacting with host FUS molecules. Large folded protein (large blue circle) diffuses slowly while being trapped inside the small voids of the droplet. In contrast, a small folded protein (small blue circle) diffuses quickly moving through the droplet voids.

The diffusion dynamics of guest proteins in FUS droplets deviate from the Stokes–Einstein relation. The diffusion coefficient is empirically represented by a power-law equation: *D* = *cM*^*α*^, where *c*, *M*, and *α* are the proportional coefficient, molecular weights of proteins, and the scaling exponent, respectively. If it follows the Stokes–Einstein relation, for example, in a dilute solution, *α* should be − 0.33^39^. In contrast, *α* changes to − 0.7 to − 0.75 in a cellular crowding condition because of the molecular sieving effect, where protein movement is slowed down by a physical entrapment inside mesh structures of cells^39,40^. For folded proteins in FUS droplets, *α*, obtained by fitting the collected data with the power-law equation, was − 1.55 ± 0.04, the absolute value of which was 4.7-fold larger than that in the Stokes–Einstein relation and twofold larger than that under cellular crowding conditions (Fig. 4A). A similar *α* value was obtained for folded proteins in p53 droplets (*α* = − 1.50 ± 0.03; Fig. 4B). A plausible scenario for these large absolute scaling exponents is shown in Fig. 4C. A large folded protein moves through relatively large voids, but cannot enter relatively small voids, which restricts the moving space only for large proteins. This is consistent with the fact that the excluded volume effect slows down the dynamics of 4E binding protein 2 within Ddx4 droplets^7^. In contrast, a small folded protein does not experience such a restriction, enabling it to move through voids of a wide size range. Therefore, the molecular sieving effect in droplets, which occurs particularly in large proteins, causes a large scaling dependence on folded proteins.

Unlike folded proteins, IDPs do not exhibit large scaling dependence on diffusion. The *α* of IDPs in FUS droplets was − 0.33 ± 0.04, the absolute value of which was 4.7-fold smaller than that of folded proteins in FUS droplets (Fig. 4A). This is consistent with the Stokes–Einstein relationship at high viscosity. A small scaling exponent was observed for IDPs in p53 droplets, which deviated from that of the Stokes–Einstein relation in the opposite direction (*α* = − 0.22 ± 0.02; Fig. 4B). These small scaling dependencies were attributed to the adaptable IDP structure. As observed in molecular dynamics simulations of p53 droplets^13^, flexible disordered regions of IDP are inserted into a highly dense region of host molecules in droplets, which enables tight interaction with neighboring host molecules, thereby slowing down the movement of IDP in droplets (Fig. 4C). In contrast to IDPs, folded proteins cannot adapt their structures to form tight interactions with host molecules. Accordingly, the enhanced intermolecular interactions of IDPs reduce the scaling dependence, in contrast to the case of folded proteins. Therefore, the different structural properties of IDPs and folded proteins cause different scaling dependences in the droplets.

In membraneless organelles, guest proteins may perform physiological functions according to their recruitment and diffusion rules. The disordered regions enable guest proteins to be concentrated in FUS droplets. In contrast, large folded proteins are excluded from the droplets. These opposite effects, as well as specific host–guest interactions, participate in guest-selective uptake into the droplets. In addition, small folded proteins recruited into droplets move quickly through voids, and may bring their ligands to other partner proteins, such as large folded proteins trapped within a large void. The contribution of recruitment and diffusion rules to physiological functions may be worth exploring.

## Methods

### FUS mutants

We prepared *human* FUS fused to MBP, 6×His-MBP-TEV-FUS, with and without a C-terminal engineered Cys, as reported previously^26^. These samples were expressed and purified without the 6×His-MBP tag cleavage. The nucleic acids in purified FUS solution, which might affect the droplet formation, was removed by washing the samples fixed to HisTrap column with 1.5 M NaCl solution.

### Guest samples

For p53 samples, we prepared the p53 tetramer as well as the NCT, TC, dimer, and monomer mutants, as described previously^11,13,34,41,42^. For the p53 tetramer, a thermostable and cysteine-modified human p53 mutant (C124A, C135V, C141V, W146Y, C182S, V203A, R209P, C229Y, H233Y, Y234F, N235K, Y236F, T253V, N268D, C275A, C277A, and K292C) was used^41^. The TC mutant corresponds to residues 293–393 of the tetramer, with an additional N-terminal cysteine^41^. The NCT mutant corresponds to residues 1–363 of the tetramer^33^. The dimer and monomer mutants corresponded to L344A and L344P of the tetramer sequence, respectively^11,13^.

The *Escherichia coli* Fis Q21C mutant and *Saccharomyces cerevisiae* Nhp6A 2-Cys mutant (containing Cys at residue 2 and the C-terminal end) were expressed and purified without tags, as described previously^43–45^. For dCas9 (deactivated Cas9 from *Streptococcus pyogenes*) samples, 10 × His-MBP-TEV-dCas9 (M1C, D10A, C80S, H840A, C574S; 60815; Addgene) was expressed and purified with or without 10 × His-MBP tag cleavage, as described previously^46^. For GFP, the opt-mutant (S30R, Y39I, F64L, S65T, F99S, N105K, E111V, I128T, Y145F, M153T, V163A, K166T, I167V, I171V, S205T, and A206V) with a C-terminal 6×His tag was expressed and purified as previously described^47^. *Lactococcus lactis* Fpg and HU containing an engineered Cys residue at the C-terminus were overproduced and purified as described previously^48,49^. For *E. coli* CRP fused to GFP, purified CRP with N-terminal eGFP was provided by Sridhar Mandali and Reid C. Johnson (UCLA).

Guest proteins having at least one disordered domain were classified into IDPs and the others were into folded proteins.

### Labeling with fluorophores

Except for CRP-GFP and GFP, the proteins were labeled with Atto488 (ATTO-TEC) (Thermo Fisher) using maleimido chemistry and then purified using a cation exchange, heparin, or gel filtration column. The N-termini of poly-R (poly-L-R with 15–70 kDa and median 200-mer; Sigma-Aldrich) and poly-D (poly-L-D with 23 kDa and 200-mer; ALAMANDA polymers) were labeled with Atto488 via succinimidyl ester chemistry and purified using gel filtration^26^.

### Recruitment measurements

Recruitment measurements were performed in accordance with the method described in our previous paper^13^. Solutions containing 10 μM non-labeled FUS-MBP, 100 mM Tris, 100 mM KCl, 1 mM dithiothreitol (DTT), 100 mg/mL dextran (MW 45,000–65,000; Sigma-Aldrich), and 100 nM fluorescent samples at pH 7.4 were used. Droplet formation was triggered by a sixfold dilution of a non-labeled FUS stock solution in dextran buffer. The solutions were then incubated at 20 °C for at least 5 min. The sample solutions were cast on a coverslip and covered with a glass slide (Matsunami Glass) using a 20-μm-thick double-sided tape. The coverslip was cleaned with a solution containing H_2_O_2_, 30% NH_3_, and H_2_O at a 1:1:1 ratio before use. An inverted fluorescence microscope (IX-73; Olympus) with a total internal reflection fluorescence unit (IX3RFAEVAW; Olympus) was used^43,44^. The objective lens (NA = 1.49) was illuminated using a 488-nm laser with highly inclined thin illumination (HILO) geometry. The fluorescence collected by the objective lens was detected using an EM-CCD camera (iXon Ultra 888; Andor). To prevent photo-bleaching of the fluorescent samples, we used a 0.15 mW laser power. The images were acquired at 20 °C. Using the ImageJ software, we calculated the average fluorescence intensities of individual droplets (*I*_droplet_) and solutions (*I*_solution_) near the droplets with background substitution and obtained EI values by dividing *I*_droplet_ by *I*_solution_.

### Single-molecule measurements

Single-molecule measurements were performed following the method described in our previous paper^13^. We used solutions containing 10 μM non-labeled FUS-MBP, 100 mM Tris, 100 mM KCl, 1 mM DTT, 100 mg/mL dextran, and 0.1–0.5 nM fluorescent samples at pH 7.4. The aforementioned microscope with HILO illumination was used to reduce the illuminated volume for single-molecule detection. The laser power was in the range of 3–5 mW. We recorded images at time intervals of 15–150 ms after reducing the number of observable molecules in the droplets by photobleaching for 1–2 min. To prevent fluorescent sample adsorption, we coated the coverslip with a 0.5% 2-methacryloyloxyethyl phosphorylcholine polymer (Lipidureμ-CM5206; NOF Corp.) in ethanol^50^. The fluorescent spots of single molecules were tracked from sequential images using the ImageJ software with the plugin ‘Particle track and analysis’. We selected trajectories with at least six consecutive points, and MSDs were calculated from all pairs of two-dimensional positions of a molecule at each time interval for all trajectories using our in-house program with some modifications^41,44^. The average *D* values were calculated by fitting the slopes of the MSD plots (five data points) with 4*D*. We calculated the *D* values for each molecule using the MSDs of the initial five displacement steps of a single molecule divided by a fourfold time interval^51,52^.

## Supplementary Information

Supplementary Information.
