# Supplementary material for: Structure-dependent recruitment and diffusion of guest proteins in liquid droplets of FUS
Source: Sci Rep. 2022 May 2;12:7101. doi: 10.1038/s41598-022-11177-w (PMC9061845; doi:10.1038/s41598-022-11177-w)
Supplement: Supplementary file 1 — Supplementary Information. [file 41598_2022_11177_MOESM1_ESM.docx]

Supplementary Information for

**Structure-dependent recruitment and diffusion of guest proteins in liquid droplets of FUS**

Kiyoto Kamagata^1,2,3*^, Nanako Iwaki^1,2^, Saori Kanbayashi^1^, Trishit Banerjee^1,2^, Rika Chiba^1,3^, Virginie Gaudon^4^, Bertrand Castaing^4^, and Seiji Sakomoto^5^

*Corresponding author: Kiyoto Kamagata

Institute of Multidisciplinary Research for Advanced Materials, Tohoku University, Katahira 2-1-1, Aoba-ku, Sendai 980-8577, Japan

TEL: +81-22-217-5843/FAX: +81-22-217-5842

e-mail: kiyoto.kamagata.e8@tohoku.ac.jp


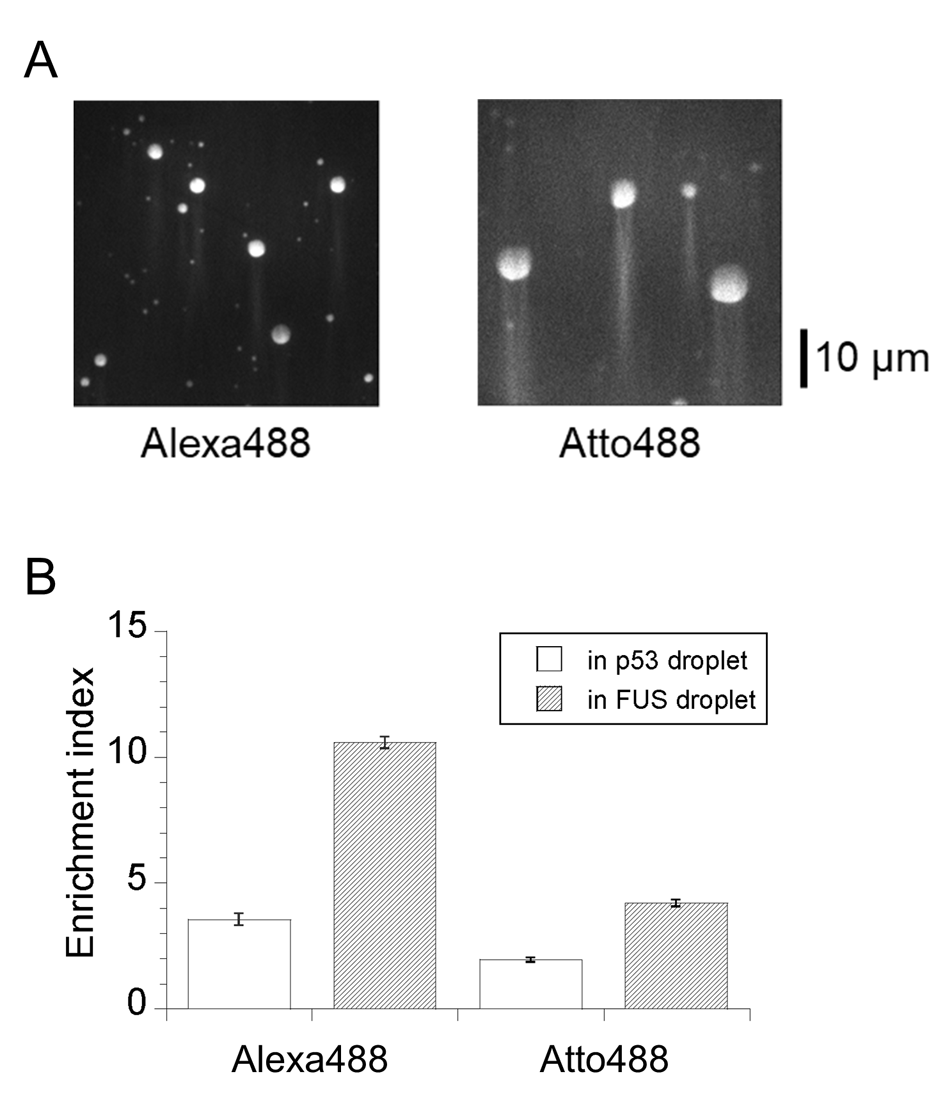


**Fig. S1.** (A) Fluorescent images of fluorescent dyes (Alexa488 and Atto488) in non-labeled FUS droplet solution. (B) Enrichment index (EI) of the fluorescent dyes into FUS and p53 droplets. The bars and errors represent the mean and standard errors for the average EI of each droplet, respectively. The EI values in p53 droplets were plotted from a reference ^1^.


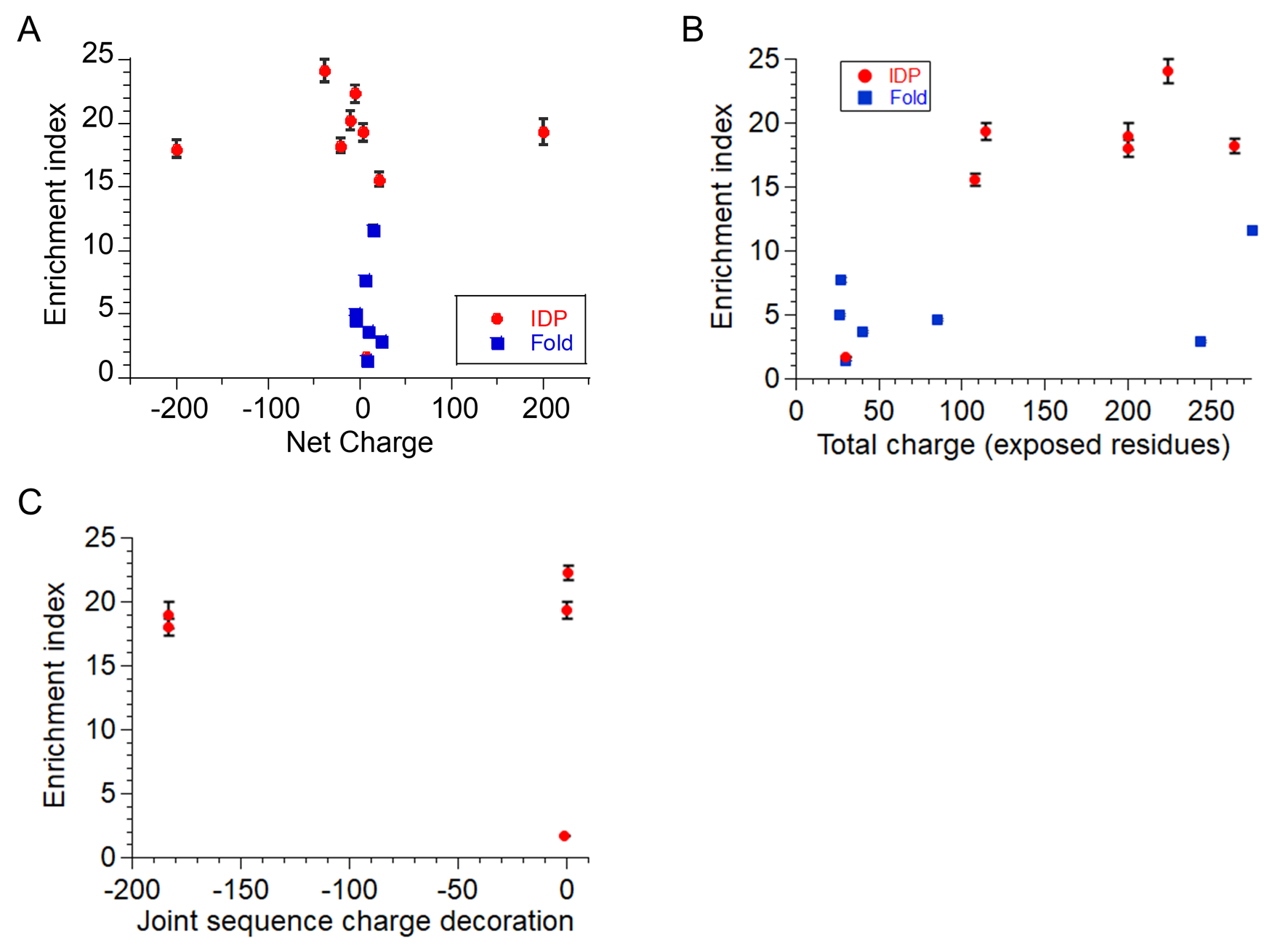


**Fig. S2.** **Enrichment index of guests in FUS droplets versus physical parameters of guest proteins.** (A) Net charge of guest proteins. (B) Total charge for solvent-exposed residues of guest proteins. Solvent-exposed residues of 3D structure of guests, including the disordered region of Np6A, were determined using GETAREA ^2^ with > 50% of the solvent accessible surface area. PDB codes used in this analysis were 2LA6 and 6G99 for RRM and ZnF domains of FUS, 1Y4C for MBP, 2OCJ and 1AIE for core and tetramerization domains of p53, 1LWM for Nhp6A, 1PJJ for Fpg, 3IV5 for FIS, 5LVT for HU, 4N9H for CRP, 4CMP for Cas9, and 1Y4C for MBP. Structure-undetermined regions in folded domains and disordered regions were assumed as solvent-exposed. (C) Joint sequence charge decoration (jSCD) ^3^ for host and guest proteins (monomeric IDPs or artificial polymers). Only disordered regions were used for the calculation of jSCD.


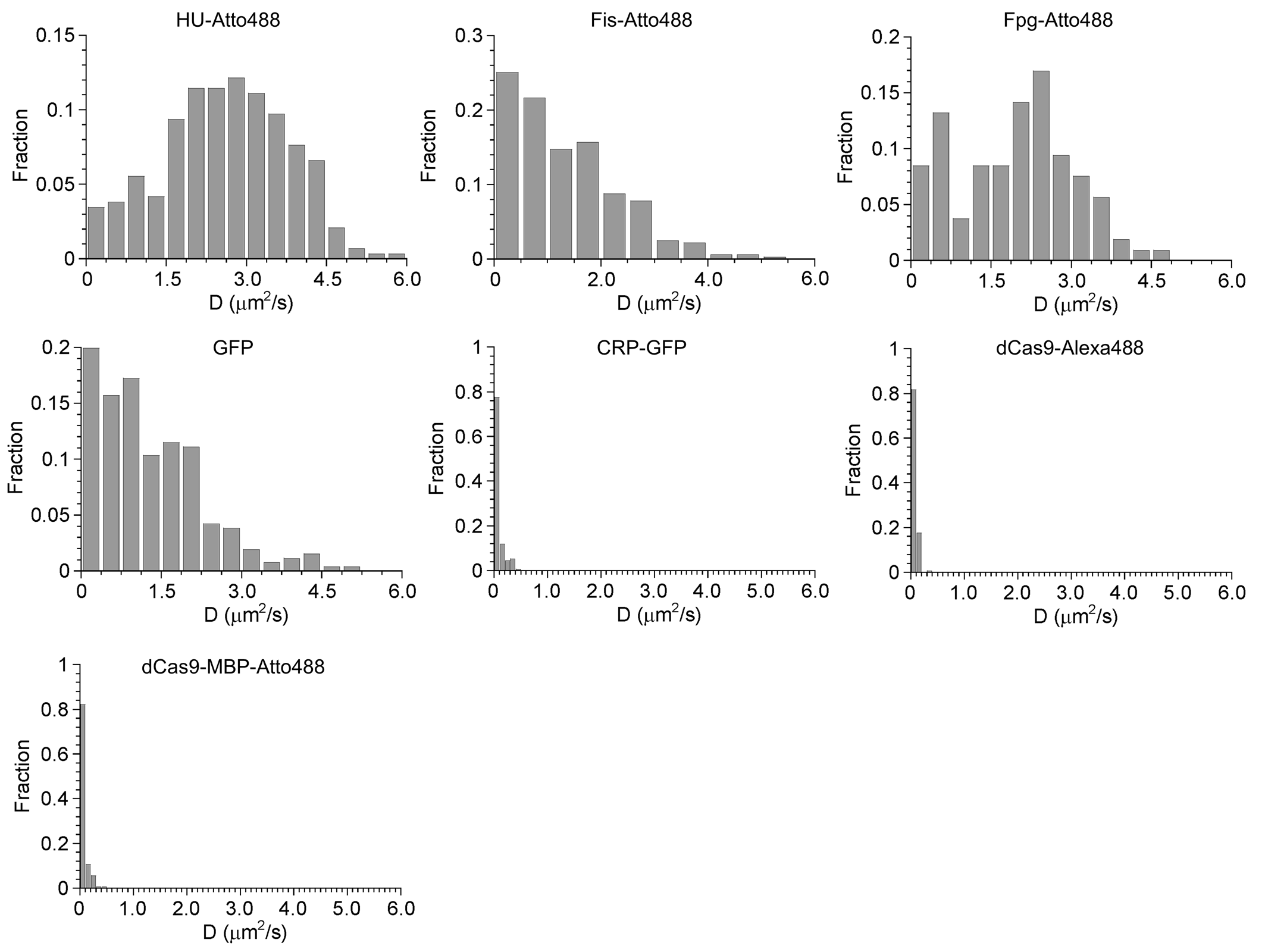


**Fig. S3.** Distribution of diffusion coefficients of individual molecules of folded proteins in FUS droplets.

References

1. Kamagata, K., et al. Molecular principles of recruitment and dynamics of guest proteins in liquid droplets. *Sci. Rep.* **11**, 19323 (2021).

2. Fraczkiewicz, R. & Braun, W. Exact and efficient analytical calculation of the accessible surface areas and their gradients for macromolecules. *J. Comput. Chem.* **19**, 319-333 (1998).

3. Amin, A. N., Lin, Y. H., Das, S. & Chan, H. S. Analytical theory for sequence-specific binary fuzzy complexes of charged intrinsically disordered proteins. *J. Phys. Chem. B* **124**, 6709-6720 (2020).
